# Supplementary material for: EDA Variants Are Responsible for Approximately 90% of Deciduous Tooth Agenesis
Source: Int J Mol Sci. 2024 Sep 27;25(19):10451. doi: 10.3390/ijms251910451 (PMC11477375; doi:10.3390/ijms251910451)
Supplement: Supplementary file 1 [file ijms-25-10451-s001.zip › Supplementary Figures.pdf]

Supplementary Figure

EDA variants are responsible for approximately 90% of deciduous tooth agenesis

Lanxin Su[1, #], Bichen Lin[2, #], Miao Yu[1], Yang Liu[1], Shichen Sun[1, 3], Hailan Feng[1], Haochen Liu[1*], Dong Han[1*]

A

Patients in this study

Gene		*EDA*	*PAX9*	*LRP6*	*MSX1*	*BMP4*	*WNT10A*	*PITX2*	*EDARADD*	Undefined
Syndrome	Number of patients (percentage)	63 (92.6%)	0	1 (1.5%)	0	1 (1.5%)	1 (1.5%)	1 (1.5%)	1 (1.5%)	0
NSTA	Number of patients (percentage)	10 (62.5%)	2 (12.5%)	1 (6.3%)	2 (12.5%)	0	0	0	0	1 (6.3%)

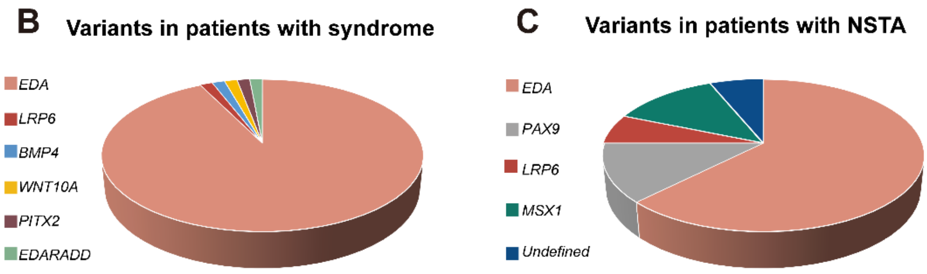

Figure S1. Variants found in the patient cohort. (A) Number and percentage of patients with syndrome or non-syndromic tooth agenesis (NSTA). (B) Proportion of different gene variants in patients with syndrome. (C) Proportion of different gene variants in patients with NSTA.

Abbreviations: *EDA, ectodysplasin A*; *EDAR, ectodysplasin A receptor*; TNF, tumor necrosis factor; TM, transmembrane
