# Supplementary material for: EDA Variants Are Responsible for Approximately 90% of Deciduous Tooth Agenesis
Source: Int J Mol Sci. 2024 Sep 27;25(19):10451. doi: 10.3390/ijms251910451 (PMC11477375; doi:10.3390/ijms251910451)
Supplement: Supplementary file 1 [file ijms-25-10451-s001.zip › Supplementary Tables.pdf]

Supplementary Tables

EDA variants are responsible for approximately 90% of deciduous tooth agenesis

Lanxin Su[1, #], Bichen Lin[2, #], Miao Yu[1], Yang Liu[1], Shichen Sun[1, 3], Hailan Feng[1], Haochen Liu[1*], Dong Han[1*]

Table S1. Dental phenotype and genetic information of patients.

Gene	Patient	Mutation	References	Jaw	Right quadrants							Left quadrants						
					7	6	5	4	3	2	1	1	2	3	4	5	6	7
EDA	1	c.170C>G	Wang et al (2003) [1]	U	*	*	*	*	*	*			*	*	*	*	*	*
		p.Thr57Arg		L	*	*	*	*	*	*	*	*	*	*	*	*	*	*
				u			*	*	*	*	#	#	*	*	*	*		
				l			*	*	*	*	*	*	*	*	*	*		
EDA	2	c.1133C>T	Wang et al (2003) [1]	U	*		*	*	*	*			*		*	*		*
		p.Thr378Met		L	*		*	*	*	*	*	*	*	*	*	*		*
				u				*	*	*	#	#	*	*	*			
				l			*	*	*	*	*	*	*	*	*	*		
EDA	3	c.959A>G	Wang et al (2003) [1]	U			*	*	#	*	*	*	*	#	*	*		
		p.Tyr320Cys		L			*	*	*	*	*	*	*	*	*	*		
				u				*	*	*	*	*	*	*	*			
				l				*	*	*	*	*	*	*	*			
EDA	4	c.1133C>T	Zhang et al (2011) [2]	U	*	*	*	*	*	*	*	*	*	*	*	*	*	*
		p.Thr378Met		L	*	*	*	*	*	*			*	*	*	*	*	*
				u	Cannot defined													
				l														

Abbreviations: *EDA*, *ectodysplasin A*; *EDAR*, *ectodysplasin A receptor*; TNF, tumor necrosis factor; TM, transmembrane

EDA	5	c.882_885del<br>p.Glu294Aspfs*12	Zhang et al (2011) [2]	U	*	*	*	*	*	*	*	*	*	*	*	*	*	*
				L	*	*	*	*	*	*	*	*	*	*	*	*	*	*
				u			*	*	*	*	*	*	*	*	*	*		
				l			*	*	*	*	*	*	*	*	*	*		
EDA	6	c.902A>G<br>p.Tyr301Cys	Zhang et al (2011) [2]	U	*		*	*	*	*	*	#	#	*	*	*		*
				L	*	*	*	*	*	*	*	*	*	*	*	*	*	*
				u	Cannot defined													
				l														
EDA	7	c.252del<br>p.Gly85Alafs*6	Zhang et al (2011) [2]	U	*	*	*	*	*	*	#	#	*	#	*	*		
				L	*	*	*	*	#	*	*	*	*	#	*	*	*	*
				u	Cannot defined													
				l														
EDA	8	c.947A>G<br>p.Asp316Gly	Zhang et al (2011) [2]	U	*	*	*	*	*	*	*	*	*	*	*	*	*	*
				L	*	*	*	*	*	*	*	*	*	*	*	*	*	*
				u			*	*	*	*	*	*	*	*	*	*		
				l			*	*	*	*	*	*	*	*	*	*		
EDA	9	c.467G>A<br>p.Arg156His	Zhang et al (2011) [2]	U			*	*	*	*	#	#	*	*	*	*		
				L	*	*	*	*	#	*	*	*	*	#	*	*	*	*
				u	Cannot defined													
				l														
EDA	10	c.467G>A<br>p.Arg156His	Zhang et al (2011) [2]	U			*	*	*	*			*	*	*	*		
				L	*	*	*	*	*	*	*	*	*	#	*	*	*	*
				u	Cannot defined													
				l														
EDA	11	c.1013C>T<br>p.Thr338Met	Han et al (2008) [3]	U				*		*	*	*	*		*			
				L			*			*	*	*	*			*		

				u	Cannot defined													
				l														
EDA	12	c.1133C>T	Zhang et al (2011) [2]	U	*		*	*	*	*			*	*	*	*	*	*
		p.Thr378Met		L	*	*	*	*	*	*	*	*	*	*	*	*		*
				u				*	#	*	#	#	*	#	*			
				l			*	*	*	*	*	*	*	*	*	*		
EDA	13	c.776C>A	Song et al (2009) [4]	U				*	*	*			*	*	*	*		
		p.Ala259Glu		L			*	*	#	*	*	*	*	#	*	*		
				u	Cannot defined													
				l														
EDA	14	c.1045G>A	Zhang et al (2011) [2]	U	*		*	*	*	#	#	#	#	*	*	*		*
		p.Ala349Thr		L			*	*	*	*	*	*	*	*	*	*		*
				u				*	*	*	#	#	*	*	*			
				l			*	*	*	*	*	*	*	*	*	*		
EDA	15	c.643G>T	Zhang et al (2011) [2]	U	*	*	*	*		*	*	*	*		*	*	*	*
		p.Gly215*		L	*	*	*	*	*	*	*	*	*	*	*	*	*	*
				u			*	*	*	*	#	*	*	*	*	*		
				l			*	*	*	*	*	*	*	*	*	*		
EDA	16	c.457C>T	Zhang et al (2011) [2]	U	*		*	*	#	*	#	#	*	#	*	*		
		p.Arg153Cys		L			*	*	#	#	*	*	#	#	*	*		
				u	Cannot defined													
				l														
EDA	17	c.106_118del	Zhang et al (2011) [2]	U	*	*	*	*	*	*	*	*	*	*	*	*	*	*
		p.Glu36Alafs*16		L	*	*	*	*	*	*	*	*	*	*	*	*	*	*
				u				*	#	*			*	#	*			
				l			*	*	#	*	*	*	*	#	*	*		

EDA	18	c.769G>C<br>p.Gly257Arg	He et al (2013) [5]	U	*		*	*	*	*			*	*	*	*		*
				L	*		*	*	*	*	*	*	*	*	*	*		*
				u				*	*	*			*	*	*			
				l				*	*	*	*	*	*	*	*			
EDA	19	c.769G>C<br>p.Gly257Arg	He et al (2013) [5]	U	*		*	*	*	*			*	*	*	*		*
				L	*		*	*	*	*	*	*	*	*	*	*		*
				u				*	*	*			*	*	*			
				l				*	*	*	*	*	*	*	*			
EDA	20	c.914G>T<br>p.Ser305Ile	He et al (2016) [6]	U	*	*	*	*	*	*	*	*	*	*	*	*	*	*
				L	*	*	*	*	*	*	*	*	*	*	*	*	*	*
				u			*	*	*	*	*	*	*	*	*	*		
				l			*	*	*	*	*	*	*	*	*	*		
EDA	21	c.602G>T<br>p.Gly201Val	He et al (2016) [6]	U	*	*	*	*	*	*			*	*	*	*	*	*
				L	*		*	*		*	*	*	*		*	*		*
				u	Cannot defined													
				l														
EDA	22	c.865C>T<br>p.Arg289Cys	This study	U	*			*	*	*	*	*	*	*	*			*
				L	*		*	*	*	*	*	*	*	*	*	*		*
				u				*	#	*		*	*	#	*			
				l					#	*	*	*	*	#				
EDA	23	c.1045G>A<br>p.Ala349Thr	He et al (2013) [5]	U	*	*	*	*	*	*	*	*	*	*	*	*	*	*
				L	*	*	*	*	*	*	*	*	*	*	*	*	*	*
				u	Cannot defined													
				l														
EDA	24	c.895G>A<br>p.Gly299Ser	This study	U	*	*	*	*	*	*			*	*	*	*	*	*
				L	*	*	*	*	*	*	*	*	*	*	*	*	*	*

				u	Cannot defined													
				l														
EDA	25	c.895G>A	This study	U	*		*	*	#	#	*	*	*	#	*	*		*
		p.Gly299Ser		L	*		*	*	*	*	*	*	*	#	*	*		*
				u	Cannot defined													
				l														
EDA	26	c.1133C>T	This study	U	*	*	*	*	*	*	#	#	*	*	*	*	*	*
		p.Thr378Met		L	*	*	*	*	*	*	*	*	*	*	*	*	*	*
				u	Cannot defined													
				l														
EDA	27	c.1133C>T	This study	U		*	*	*	*	*		*	*	*	*	*	*	
		p.Thr378Met		L	*	*	*	*	*	*	*	*	*	*	*	*	*	*
				u	Cannot defined													
				l														
EDA	28	c.871G>A	This study	U	*	*	*	*	*	*	*	*	*	*	*	*	*	*
		p.Gly291Arg		L	*	*	*	*	*	*	*	*	*	*	*	*	*	*
				u			*	*	*	*	*	*	*	*	*	*		
				l			*	*	*	*	*	*	*	*	*	*		
EDA	29	c.936C>G	He et al (2013) [5]	U			*	*	*	*		*	*	*	*	*		
		p.Ile312Met		L			*	*		*	*	*	*		*	*		
				u	Cannot defined													
				l														
EDA	30	c.133G>C	He et al (2016) [6]	U				*	*	*			*	*	*			
		p.Gly45Arg		L	*			*	*	*	*	*	*	*	*			
				u	Cannot defined													
				l														

EDA	31	c.88-89insG	He et al (2016) [6]	U	*	*	*	*		*	*	*	*		*	*	*	*
		p.Ala30Glyfs*99		L	*	*	*	*	*	*	*	*	*	*	*	*	*	*
				u	Cannot defined													
				l														
EDA	32	c.28del	This study	U	*	*	*	*	*	*	#	*	*	*	*	*	*	*
		p.Glu10Asnfs*47		L	*	*	*	*	*	*	*	*	*	*	*	*	*	*
				u	Cannot defined													
				l														
EDA	33	c.916C>T	He et al (2016) [6]	U	*	*	*	*	*	*	*	*	*	*	*	*	*	*
		p.Gln306*		L	*	*	*	*	*	*	*	*	*	*	*	*	*	*
				u				*	*	*	*	*	*	*	*			
				l			*	*	*	*	*	*	*	*	*	*		
EDA	34	c.463C>T	This study	U			*	*	#	*	*	*	*	#	*	*		
		p.Arg155Cys		L			*	*	*	*	*	*	*	*	*	*		
				u	Cannot defined													
				l														
EDA	35	c.895G>A	This study	U	*	*	*	*	*	*	*	*	*	*	*	*	*	*
		p.Gly299Ser		L	*	*	*	*	*	*	*	*	*	*	*	*	*	*
				u			*	*	*	*	*	*	*	*	*	*		
				l			*	*	*	*	*	*	*	*	*	*		
EDA	36	c.1013C>T	This study	U				*	*	*			*	*	*			
		p.Thr338Met		L					*	*	*	*	*	*				
				u	Cannot defined													
				l														
EDA	37	c.656C>A; c.661G>C	This study	U	*	*	*	*	*	*	*		*	*	*	*	*	*
		p.Pro219His; p.Gly221Arg		L	*	*	*	*	*	*	*	*	*	*	*	*	*	*

				u	Cannot defined													
				l														
EDA	38	c.914G>A	This study	U	*		*	*	*	*	*	*	*	#	*	*		*
		p.Ser305Asn		L	*		*	*	*	*	*	*	*	*	*	*		*
				u				*	*	*	*	*	*	*	*			
				l			*	*	*	*	*	*	*	*	*	*		
EDA	39	c.463C>T	This study	U	*		*	*		*			*		*	*		*
		p.Arg155Cys		L	*		*	*	*	*	*	*	*		*	*		*
				u	Cannot defined													
				l														
EDA	40	c.463C>T	This study	U			*	*		*	*	*	*		*	*		
		p.Arg155Cys		L			*	*		*	*	*	*		*	*		
				u				*	#	*	#	#	*	#	*			
				l				*	#	*	*	*	*	#	*			
EDA	41	c.457C>T	This study	U	*		*	*		*			*	*	*	*		*
		p.Arg153Cys		L			*	*		*		*	*		*	*		*
				u					#	*	#	#	*	#				
				l					#	#	*	*	#	#				
EDA	42	c.673C>T; c.676C>T	Wu et al (2020) [7]	U	*	*	*	*	*	*			*	*	*	*	*	*
		p.Pro225Ser; P.Gln226*		L	*		*	*	*	*	*	*	*	*	*	*	*	*
				u			*	*	*	*	#	#	*	*	*	*		
				l			*	*	*	*	*	*	*	*	*	*		
EDA	43	c.511A>T	This study	U	*		*	*	*	*			*	*	*	*		*
		p.Lys171*		L			*	*		*	*	*	*	*	*	*		
				u	Cannot defined													
				l														

EDA	44	c.730C>T	This study	U	*	*	*	*	*	*	*	*	*	*	*	*	*	*
		p.Arg244*		L	*	*	*	*	*	*	*	*	*	*	*	*	*	*
				u	Cannot defined													
				l														
EDA	45	c.1133C>T	This study	U	*		*	*		*	*	*	*	*	*	*		*
		p.Thr378Met		L	*	*	*	*	*	*	*	*	*	*	*	*	*	*
				u			*	*	*	*	*	*	*	*	*	*		
				l			*	*	*	*	*	*	*	*	*	*		
EDA	46	c.1133C>T	This study	U	*		*	*	*	*			*	*	*	*		*
		p.Thr378Met		L	*	*	*	*	*	*	*	*	*		*	*	*	*
				u				*	*	*	*	*	#	#	*			
				l			*	*	#	*	*	*	*	#	*	*		
EDA	47	c.1133C>T	This study	U	*	*	*	*	*	*	*	*	*	*	*	*	*	*
		p.Thr378Met		L	*	*	*	*	*	*	*	*	*	*	*	*	*	*
				u			*	*	*	*	*	*	*	*	*	*		
				l			*	*	*	*	*	*	*	*	*	*		
EDA	48	c.983C>G	This study	U			*	*	*	*	#	#	*	*	*	*		*
		p.Pro328Arg		L	*			*		*	*	*	*		*			*
				u				*		*			*		*			
				l				*		*	*	*	*		*			
EDA	49	c.457C>T	Wu et al (2020) [7]	U			*	*	*	*	#	#	*	*	*	*		
		p.Arg153Cys		L			*	*		*	*	*	*		*	*		
				u				*	#	*			*	#	*			
				l				*	#	*	*	*	*	#	*			
EDA	50	c.905T>G	Wu et al (2020) [7]	U			*		*	*			*	*		*		
		p.Phe302Cys		L				*	*	*	*	*	*	*				

				u					*	*	#	#	*	*				
				l					*	*	*	*	*	*				
EDA	51	c.466C>T p.Arg156Cys	Wu et al (2020) [7]	U	*	*	*	*	*	*	*	*	*	*	*	*	*	*
				L	*	*	*	*	*	*	*	*	*	*	*	*	*	
				u				*	*	*	*	*	*	*	*	*		
				l			*	*	*	*	*	*	*	*	*	*		
EDA	52	c.1013C>T p.Thr338Met	This study	U	*		*	*	*	*	*	*	*	*	*	*		*
				L	*		*		*	*	*	*	*	*	*			*
				u				*	*	*	*	*	*	*	*			
				l					*	*	*	*	*	*				
EDA	53	c.164T>C p.Leu55Pro	Wu et al (2020) [7]	U	*	*	*	*	*	*			*	*	*	*	*	*
				L	*	*	*	*	*	*	*	*	*	*	*	*	*	*
				u			*	*	*	*	#	#	*	*	*	*		
				l			*	*	*	*	*	*	*	*	*	*		
EDA	54	c.164T>C p.Leu55Pro	Wu et al (2020) [7]	U	*	*	*	*	*	*			*	*	*	*	*	*
				L	*	*	*	*	*	*	*	*	*	*	*	*		*
				u	Cannot defined													
				l														
EDA	55	c.619del p.Gly207Glufs*73	Wu et al (2020) [7]	U	*		*	*		*	#	#	*		*	*		*
				L	*	*	*	*	*	*	*	*	*		*	*	*	*
				u				*	#	*		*	*	#	*			
				l			*	*	#	*	*	*	*	#	*	*		
EDA	56	c.457C>T p.Arg153Cys	Wu et al (2020) [7]	U			*	*	*	*			*	*	*	*		
				L			*		*	*		*	*	*	*	*		
				u	Cannot defined													
				l														

EDA	57	c.463C>T p.Arg155Cys	This study	U	*		*	*		*			*		*	*		*
				L				*	*	*	*	*	*	*	*			
				u	Cannot defined													
				l														
EDA	58	c.583G>A p.Gly195Arg	This study	U	*	*	*	*	*	*	*	*	*	*	*	*	*	*
				L	*	*	*	*	*	*	*	*	*	*	*	*	*	*
				u			*	*	*	*	*	*	*	*	*	*		
				l			*	*	*	*	*	*	*	*	*	*		
EDA	59	c.871G>A p.Gly291Arg	This study	U	*		*	*	*	*	*	*	*	*	*	*		*
				L	*	*	*	*	*	*	*	*	*	*	*	*	*	*
				u			*	*	*	*	*	*	*	*	*	*		
				l			*	*	*	*	*	*	*	*	*	*		
EDA	60	c.1045G>A p.Ala349Thr	This study	U	*	*	*	*	*	*	*	*	*	*	*	*	*	*
				L	*	*	*	*	*	*	*	*	*	*	*	*	*	*
				u				*	#	*	*	*	*	*	*			
				l			*	*	*	*	*	*	*	*	*	*		
EDA	61	c.467G>A p.Arg156His	This study	U	*		*	*	*	*	#	#	*	*	*	*		*
				L	*		*	*	*	*	*	*	*	*	*	*		*
				u				*	*	*	#	#	*	*	*			
				l			*	*	*	*	*	*	*	*	*	*		
EDA	62	c.572dup p.Gly192Argfs*48	This study	U	*	*	*	*	*	*	*	*	*	*	*	*	*	*
				L	*	*	*	*	*	*	*	*	*	*	*	*	*	*
				u			*	*	*	*	*	*	*	*	*	*		
				l			*	*	*	*	*	*	*	*	*	*		
EDA	63	c.584G>A p.Gly195Glu	Wu et al (2020) [7]	U	*	*	*	*	*	*			*	*	*	*	*	*
				L	*	*	*	*		*	*	*	*	*	*	*	*	*

				u			*	*	*	*	#	*	*	*	*	*		
				l			*	*	*	*	*	*	*	*	*	*		
EDA	64	c.457C>T	This study	U	*			*		*			*		*			*
		p.Arg153Cys		L	*			*			*	*	*		*			*
				u	Cannot defined													
				l														
EDA	65	c.584G>A	This study	U	*		*	*	*		*			*	*	*		*
		p.Gly195Glu		L	*	*	*	*	*	*	*	*	*	*	*	*	*	*
				u			*	*	*	*			*	*	*	*		
				l			*	*	*	*	*	*	*	*	*	*		
EDA	66	c.584G>A	This study	U	*	*	*	*	*	*			*	*	*	*	*	*
		p.Gly195Glu		L	*	*	*	*	*	*			*	*	*	*	*	*
				u			*	*	#	*	#	#	*	#	*	*		
				l			*	*	#	*	*	*	*	#	*	*		
EDA	67	c.781dup	This study	U	*		*	*	*	*	*	*	*	*	*	*		*
		p.Gln261Profs*5		L	*		*	*	*	*	*	*	*	*	*	*	*	*
				u				*	#	*	#	#	*	#	*			
				l			*	*	#	*	*	*	*	#	*	*		
EDA	68	c.1013C>T	This study	U	*		*		*	*	*	*	*	*		*		
		p.Thr338Met		L			*		*	*	*	*	*	*		*		
				u						*	*	*	*					
				l						*	*	*	*					
EDA	69	c.463C>T	This study	U	*		*	*	*	*			*	*	*	*		*
		p.Arg155Cys		L	*		*	*	*	*	*	*	*	*	*	*		*
				u				*	*	*	#	#	*	*	*			
				l				*	*	*	*	*	*	*	*			

EDA	70	c.502+1G>A	This study	U	*		*	*	*	*	*	*	*	*	*	*		*
				L	*		*	*	*	*	*	*	*	*	*	*		*
				u				*	#	*	*	*	*	#	*			
				l				*	#	*	*	*	*	#	*			
EDA	71	c.467G>A<br>p.Arg156His	This study	U	*		*	*		*			*		*	*		*
				L	*		*	*		*	*	*	*		*	*		*
				u	Cannot defined													
				l														
EDA	72	c.511A>T<br>p.K171*	This study	U	*	*	*	*		*			*		*	*	*	*
				L	*	*	*	*	*	*	*	*	*	*	*	*	*	*
				u	Cannot defined													
				l														
EDA	73	c.1013C>T<br>p.Thr338Met	This study	U			*	*	*	*			*	*	*	*		
				L			*	*		*	*	*	*	*	*	*		
				u				*		*			*	*	*			
				l						*	*	*	*					
PAX9	74	c.236_237insAC<br>p.Thr80Leufs*6	Sun et al (2021) [8]	U	*	*	*	*			#	#	*			*	*	*
				L	*	*	*				*	*				*	*	*
				u			*									*		
				l			*									*		
PAX9	75	c.336C>A<br>p.Cys112*	This study	U	*	*	*		*	*				*		*	*	*
				L	*	*	*									*	*	*
				u	Cannot defined													
				l														
LRP6	76	c.2292G>A<br>p.Trp764*	Yu et al (2021) [9]	U	*	*	*	*		*			*		*	*	*	*
				L			*	*		*	*	*	*		*	*		

				u			*	*							*	*		
				l				*							*			
LRP6	77	c.716G>A p.Trp239*	This study	U						*			*					
				L						*	*	*	*					
				u														
				l						*	*	*	*					
MSX1	78	c.670C>T p.Arg224Cys	Zheng et al (2021) [10]	U			*	*		*	*	*	*		*	*		
				L	*	*	*			*	*	*				*	*	*
				u														
				l			*									*		
MSX1	79	c.421del p.Glu141Argfs*19	This study	U			*	*		*			*		*	*		
				L	*	*	*			*	*	*				*	*	*
				u			*									*		
				l			*									*		
BMP4	80	c.614T>C p.Val205Ala	Yu et al (2019) [11]	U	*	*	*		*	*				*		*	*	*
				L	*	*	*									*	*	*
				u	Cannot defined													
				l														
WNT10A	81	c.826T>A; c.949del p.Cys276Ser p.Ala317Hisfs*121	Yu et al (2019) [12]	U	*	*	*	*	*	*	*	*	*	*	*	*	*	*
				L	*	*	*	*	*	*	*	*	*	*	*	*	*	*
				u						*			*					
				l						#	#	#	#					
PITX2	82	c.630insCG p.Val211Argfs*28	Fan et al (2019) [13]	U	*	*	*	*	*	*	*	*	*	*	*	*		*
				L	*		*			*	*	*	*	*	*	*		
				u			*	*		*	*	*	*	*	*	*		

				l														
EDARADD	83	c.208_209insAGAATAATTTC p.Met70Lysfs*5	This study	U	*	*	*	*	*	*			*	*	*	*	*	*
				L	*	*	*	*	*	*	*	*	*	*	*	*	*	*
				u			*	*	*	*	#	#	*	*	*	*		
				l			*	*	*	*	*	*	*	*	*	*		
Undefined	84	Undefined	This study	U			*	*	*	*	*	*	*	*	*			
				L			*	*	*	*	*	*	*	*	*	*		
				u			*	*	*	*	*	*	*	*	*	*		
				l			*	*	*	*	*	*	*	*	*	*		

Asterisks (*) mark the missing teeth. Pound (#) keys mark the mal-formed teeth. The references in the table cited the patients who have published by our team in the past.

References

1. Wang, Y.; Zhao, H.; Zhang, X.; Feng, H. [Mutation detection in ED1 gene in hypohidrotic ectodermal dysplasia (HED) families]. *Beijing Da Xue Xue Bao Yi Xue Ban* **2003**, *35*, 419-422.
2. Zhang, J.; Han, D.; Song, S.; Wang, Y.; Zhao, H.; Pan, S.; Bai, B.; Feng, H. Correlation between the phenotypes and genotypes of X-linked hypohidrotic ectodermal dysplasia and non-syndromic hypodontia caused by ectodysplasin-A mutations. *Eur J Med Genet* **2011**, *54*, e377-382, doi:10.1016/j.ejmg.2011.03.005.
3. Han, D.; Gong, Y.; Wu, H.; Zhang, X.; Yan, M.; Wang, X.; Qu, H.; Feng, H.; Song, S. Novel EDA mutation resulting in X-linked non-syndromic hypodontia and the pattern of EDA-associated isolated tooth agenesis. *Eur J Med Genet* **2008**, *51*, 536-546, doi:10.1016/j.ejmg.2008.06.002.
4. Song, S.; Han, D.; Qu, H.; Gong, Y.; Wu, H.; Zhang, X.; Zhong, N.; Feng, H. EDA gene mutations underlie non-syndromic oligodontia. *J Dent Res* **2009**, *88*, 126-131, doi:10.1177/0022034508328627.
5. He, H.; Han, D.; Feng, H.; Qu, H.; Song, S.; Bai, B.; Zhang, Z. Involvement of and interaction between WNT10A and EDA mutations in tooth agenesis cases in the Chinese population. *PLoS One* **2013**, *8*, e80393, doi:10.1371/journal.pone.0080393.
6. He, H.Y.; Liu, Y.; Han, D.; Liu, H.C.; Bai, B.J.; Feng, H.L. [EDA mutation screening and phenotype analysis in patients with tooth agenesis]. *Beijing Da Xue Xue Bao Yi Xue Ban* **2016**, *48*, 686-691.
7. Wu, J.Y.; Yu, M.; Sun, S.C.; Fan, Z.Z.; Zheng, J.L.; Zhang, L.T.; Feng, H.L.; Liu, Y.; Han, D. [Detection of EDA gene mutation and phenotypic analysis in patients with hypohidrotic ectodermal dysplasia]. *Beijing Da Xue Xue Bao Yi Xue Ban* **2020**, *53*, 24-33, doi:10.19723/j.issn.1671-167X.2021.01.005.

8. Sun, K.; Yu, M.; Yeh, I.; Zhang, L.; Liu, H.; Cai, T.; Feng, H.; Liu, Y.; Han, D. Functional study of novel PAX9 variants: The paired domain and non-syndromic oligodontia. *Oral Dis* **2021**, *27*, 1468-1477, doi:10.1111/odi.13684.
9. Yu, M.; Fan, Z.; Wong, S.W.; Sun, K.; Zhang, L.; Liu, H.; Feng, H.; Liu, Y.; Han, D. Lrp6 Dynamic Expression in Tooth Development and Mutations in Oligodontia. *J Dent Res* **2021**, *100*, 415-422, doi:10.1177/0022034520970459.
10. Zheng, J.; Yu, M.; Liu, H.; Cai, T.; Feng, H.; Liu, Y.; Han, D. Novel MSX1 variants identified in families with nonsyndromic oligodontia. *Int J Oral Sci* **2021**, *13*, 2, doi:10.1038/s41368-020-00106-0.
11. Yu, M.; Wang, H.; Fan, Z.; Xie, C.; Liu, H.; Liu, Y.; Han, D.; Wong, S.W.; Feng, H. BMP4 mutations in tooth agenesis and low bone mass. *Arch Oral Biol* **2019**, *103*, 40-46, doi:10.1016/j.archoralbio.2019.05.012.
12. Yu, M.; Liu, Y.; Liu, H.; Wong, S.W.; He, H.; Zhang, X.; Wang, Y.; Han, D.; Feng, H. Distinct impacts of bi-allelic WNT10A mutations on the permanent and primary dentitions in odonto-onycho-dermal dysplasia. *Am J Med Genet A* **2019**, *179*, 57-64, doi:10.1002/ajmg.a.60682.
13. Fan, Z.; Sun, S.; Liu, H.; Yu, M.; Liu, Z.; Wong, S.W.; Liu, Y.; Han, D.; Feng, H. Novel PITX2 mutations identified in Axenfeld-Rieger syndrome and the pattern of PITX2-related tooth agenesis. *Oral Dis* **2019**, *25*, 2010-2019, doi:10.1111/odi.13196.

Table S2. Symptoms of ectodermal abnormalities in patients with X-linked hypohidrotic ectodermal dysplasia (XLHED).

Clinical Features												
Patients with XLHED	Hair		Gland				Skin				Others
	Sparse hair	Sparse eyebrows	Hypohidrosis	Anhidrosis	Xerostomia	Dry eyes	Dry skin	facial erythema	Periocular hyperpigmentation	Eczema	
1	+	+		+				+	red		
2	+	+		+			+		+		
3		+	+				+			+	
4	+			+							
5	+			+	+	+					palmoplantar keratosis
6	+		+								
7		+							+		
8	+		+				+				
9	+		+		+						
10	+	+	+		+				+		
12	+	+		+	+		+				
14	+		+								
15	+			+	+		+			+	thin nails
16		+									
17							+				
20	+	+	+								+
21	+			+			+		red		desquamation
22	+										
23	+					+	+				desquamation
24	+			+	+						thin nails

25	+		+		+					
26	+		+		+					
27	+					+	+			
28	+		+							
31	+			+	+	+				
32	+	+		+	+					
33	+			+		+			+	
34			+				+			
35	+			+	+					nasal collapse
37	+	+		+	+	+		+, perioral and periocular	+	
38	+					+				
39	+		+							
40	+	+	+		+					
41		+	+							
42	+	+	+							
43										thin nails
44	+	+		+						
45		+		+					+	
46	+		+			+				
47	+		+						+	
48	curly								+	
49	+		+							dysplasia of nipple, areola and pectoralis major

50	+								
51	+		+		+	+		+	
53	+	+	+			+		+	
54	+	+	+						
55	+			+	+				
56		+	+						
57	+, curly	+	+				+		palmoplantar keratosis
58	+			+	+				
59	+		+						
60	+			+	+	+			
61		+		+	+			+	
62	+		+			+	+	+	
63	+								palmoplantar keratosis
64	+		+						
65	+			+					thin nails, nasal collapse
66		+							Kawasaki disease
67	+			+	+				
68	curly								
69	+		+			+			
70	+	+	+						
71			+						
72	+		+						palmoplantar keratosis
73	curly								

Table S3. Primers used for PCR amplification and the PCR conditions used in this study.

Gene	Exon		Primer (5′-3′)	Annealing T (°C)
EDA (NM_001399.5)	1	Forward	CAAGAGAGTGGGTGTCTCCG	55°C
		Reverse	GGAGTCTGGATTAGCTAAAC	
	2	Forward	ACTGAGTGGGGTCAACCTTT	55°C
		Reverse	TGGGTCTGTGGTGGACAGTAT	
	3	Forward	TTGGATCCTTGCCAAAAGCC	55°C
		Reverse	AACAAGGAAGAATGAAAGAGGTGA	
	4	Forward	ACAGTACACTCATCACAGGAAAT	55°C
		Reverse	AGTCAGGGAGGGCTTGTAAC	
	5	Forward	GGGCTTGCCTTGGGCTAATA	55°C
		Reverse	GAGGGGGCTGTGAGTGAAAA	
	6	Forward	TGGAAACATGGGACTGGTGG	55°C
		Reverse	TATTTGGAGGCTGGGGAGGA	
	7	Forward	TGCTGCTGATAAAGCTGGAACTC	55°C
		Reverse	CCATCTTGACGGCGATCTTCT	
	8	Forward	GGAACTCGAGAAAACCAGCCAG	55°C
		Reverse	GCTGCAACACCAATACACCTC	
PAX9 (NM_001372076.1)	2	Forward	GGACAGCCCCAGTAGTTAGTA	55°C
		Reverse	AAAGGAAAAAGCCTCAGGTGG	
MSX1 (NM_002448.3)	1	Forward	GCCACTCGGTGTCAAAGTG	55°C
		Reverse	ACATCGGTGTCCTATCCGTT	
LRP6 (NM_002336.3)	4	Forward	TTGGGGCTTTTGGACACCTT	55°C
		Reverse	GATAGCCCTCCCTCCTCCT	
